# 2-[(1,5-Dimethyl-3-oxo-2-phenyl-2,3-dihydro-1*H*-pyrazol-4-yl)amino]-1-methyl-2-oxoethyl pyrrolidine-1-carbodithio­ate

**DOI:** 10.1107/S1600536810045654

**Published:** 2010-11-13

**Authors:** Mehmet Akkurt, Nuray Ulusoy Güzeldemirci, Nesrin Cesur, Orhan Büyükgüngör

**Affiliations:** a Department of Physics, Faculty of Arts and Sciences, Erciyes University, 38039 Kayseri, Turkey; b Department of Pharmaceutical Chemistry, Faculty of Pharmacy, Istanbul University, 34116 Istanbul, Turkey; c Department of Physics, Faculty of Arts and Sciences, Ondokuz Mayıs University, 55139 Samsun, Turkey

## Abstract

In the title compound, C_19_H_24_N_4_O_2_S_2_, inversion-related mol­ecules are linked together to form a dimer by N—H⋯O and C—H⋯O hydrogen bonds, generating two *R*_2_^1^(6) rings and one *R*_2_^2^(10) ring motif. An inter­molecular C—H⋯O hydrogen bond connects the dimers to each other. An intra­molecular C—H⋯O inter­action occurs. In the pyrrolidine ring, the two C atoms of the ring not bonded to the N atom displays positional disorder with site-occupation factors of 0.630 (18) and 0.370 (18).

## Related literature

For general background to and applications of dithio­carbamate derivatives, see: Bayrak *et al.* (2010 ▶); Chourasia & Tyagi (1999 ▶); Günay *et al.* (1999 ▶); Gürsoy *et al.* (2000 ▶); Güzel & Salman (2006 ▶); Sondhi *et al.* (2001 ▶); İsmail *et al.* (2007 ▶). For reference bond-length data, see: Allen *et al.* (1987 ▶). For graph-set notation, see: Bernstein *et al.* (1995 ▶).
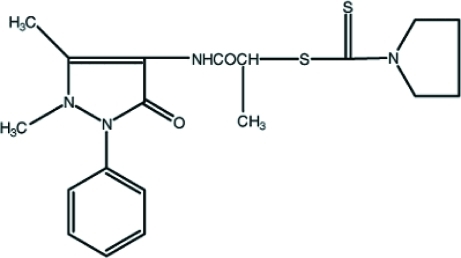


## Experimental

### 

#### Crystal data

C_19_H_24_N_4_O_2_S_2_
*M* *_r_* = 404.56
Monoclinic, 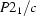

*a* = 12.0652 (5) Å
*b* = 16.0831 (6) Å
*c* = 11.0438 (4) Å
β = 101.899 (3)°
*V* = 2096.96 (14) Å^3^
*Z* = 4
Mo *K*α radiation
μ = 0.28 mm^−1^
*T* = 296 K
0.53 × 0.39 × 0.18 mm

#### Data collection

Stoe IPDS 2 diffractometer
Absorption correction: integration (*X-RED32*; Stoe & Cie, 2002 ▶) *T*_min_ = 0.880, *T*_max_ = 0.952
24440 measured reflections
4352 independent reflections
3699 reflections with *I* > 2σ(*I*)
*R*_int_ = 0.042

#### Refinement

*R*[*F* ^2^ > 2σ(*F* ^2^)] = 0.040
*wR*(*F* ^2^) = 0.121
*S* = 1.05
4352 reflections
266 parameters
6 restraints
H-atom parameters constrained
Δρ_max_ = 0.45 e Å^−3^
Δρ_min_ = −0.24 e Å^−3^

### 

Data collection: *X-AREA* (Stoe & Cie, 2002 ▶); cell refinement: *X-AREA*; data reduction: *X-RED32* (Stoe & Cie, 2002 ▶); program(s) used to solve structure: *SIR97* (Altomare *et al.*, 1999 ▶); program(s) used to refine structure: *SHELXL97* (Sheldrick, 2008 ▶); molecular graphics: *ORTEP-3* (Farrugia, 1997 ▶); software used to prepare material for publication: *WinGX* (Farrugia, 1999 ▶).

## Supplementary Material

Crystal structure: contains datablocks global, I. DOI: 10.1107/S1600536810045654/hg2741sup1.cif

Structure factors: contains datablocks I. DOI: 10.1107/S1600536810045654/hg2741Isup2.hkl

Additional supplementary materials:  crystallographic information; 3D view; checkCIF report

## Figures and Tables

**Table 1:** Hydrogen-bond geometry (Å, °)

*D*—H⋯*A*	*D*—H	H⋯*A*	*D*⋯*A*	*D*—H⋯*A*
N3—H3*A*⋯O1^i^	0.86	1.99	2.8463 (17)	171
C10—H10*A*⋯O2	0.96	2.48	3.054 (3)	119
C10—H10*C*⋯O2^ii^	0.96	2.32	3.180 (3)	148
C13—H13⋯O1^i^	0.98	2.43	3.260 (2)	143

Symmetry codes: (i) 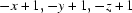; (ii) 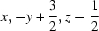.

**Fig. 1.**
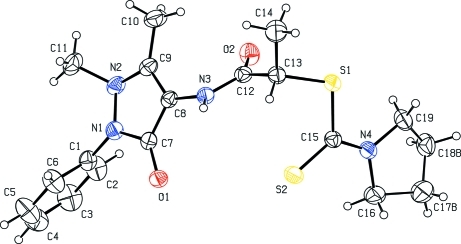
Molecule with the major component of the disorder. Displacement ellipsoids for non-H atoms are drawn at the 30% probability level.

**Fig. 2.**
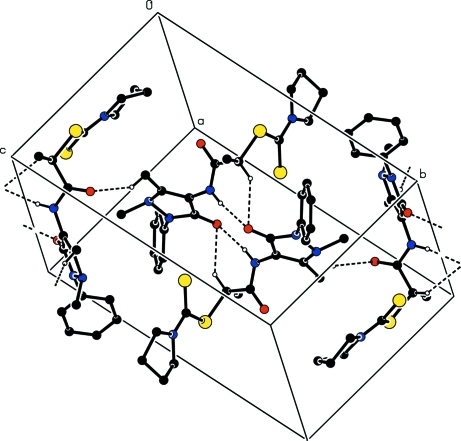
View of the dimer interactions between the neighbouring molecules with the major component of the disorder. All H atoms not involved in hydrogen bonding are omitted for clarity.

## supplementary crystallographic information

### Comment

Antipyrine (1,5-dimethyl-2-phenyl-2,3-dihydro-1*H*-pyrazol-3-one) was the first pyrazolone derivative used in the management of pain and inflammation, and their derivatives have attracted the attention of several research groups due to their potential activities. In this context, broad spectra of bioactive antipyrine derivatives have been investigated and diversities of bioactivities such as analgesic (Gürsoy *et al.*, 2000), anti-inflammatory (İsmail *et al.*, 2007), anticancer (Sondhi *et al.*, 2001) and antimicrobial activity (Bayrak *et al.*, 2010) have been reported. On the other hand, the dithiocarbamate derivatives have been reported to exhibit antibacterial (Chourasia & Tyagi, 1999), antifungal (Günay *et al.*, 1999), antimycobacterial and antitumor activities (Güzel & Salman, 2006). In view of these observations, we have synthesized the title molecule, (I), (Fig. 1), and we report here its crystal structure.

In the title compound (I), (Fig. 1), all bond lengths and angles are in normal ranges (Allen *et al.*, 1987). The dihedral angle between the connected six- and five-membered rings (C1–C5) and (N1/N2/C7–C9) is 71.68 (11)°.

In the disorder pyrrolidine ring of (I), each component is not planar [the puckering parameters: Q(2) = 0.429 (9) Å, φ(2) = 89.9 (6) ° for major component N4/C16/C17B/C18B/C19, and Q(2) = 0.375 (19) Å, φ(2) = 271.9 (13) ° for minor component N4/C16/C17A/C18A/C19].

The molecular conformation of (I) is stabilized by the intramolecular C—H···O and C—H···S interactions. The molecules are linked together to form a dimer by N3—H3A···O1^i^ and C13—H13···O1^i^ hydrogen bonds [symmetry code: (i) -*x* + 1, -*y* + 1, -*z* + 1] (Table 1 and Fig. 2), producing two *R*_2_^1^(6) rings and one *R*_2_^2^(10) ring motif (Bernstein *et al.*, 1995). An intermolecular C10—H10C···O2^ii ^hydrogen bond [symmetry code: (ii)*x*, -*y* + 3/2, *z* - 1/2] (Table 1) also connects the dimers to each other.

### Experimental

The ethanolic solution of 4-(α-chloropropionyl)amino-1,5-dimethyl-3-oxo-2-phenyl-2,3-dihydro-1*H*-pyrazole (0.005 mol) and *N,N*-disubstituted dithiocarbamate (0.005 mol) was refluxed for 1 h. After evaporation of the solvent *in vacuo*, product was washed with water and purified by recrystallization from ethanol.

Yield (%): 85. *M*.p. (*^o^*C): 158–160. IR [υ, cm^-1^, KBr]: 1684 (amide C=O), 1632 (pyrazolone C=O), 1250 (C=S). Analysis calculated for C_19_H_24_N_4_O_2_S_2_: C 56.41, H 5.97, N13.84%. Found: C 57.36, H 6.17, N 13.87%.

### Refinement

All H atoms were positioned geometrically with N—H = 0.86 Å, C—H = 0.93–0.97 Å and refined using a riding model with *U*_iso_(H) = 1.2 or 1.5*U*_eq_(C,*N*). In the pyrrolidine ring, the two C atoms of the ring not bonded to the N atom displays positional disorder with site-occupation factors of 0.630 (18) and 0.370 (18).

### Crystal data

**Table:**

C_19_H_24_N_4_O_2_S_2_	*F*(000) = 856
*M**_r_* = 404.56	*D*_x_ = 1.281 Mg m^−^^3^
Monoclinic, *P*2_1_/*c*	Mo *K*α radiation, λ = 0.71073 Å
Hall symbol: -P 2ybc	Cell parameters from 34646 reflections
*a* = 12.0652 (5) Å	θ = 1.7–28.1°
*b* = 16.0831 (6) Å	µ = 0.28 mm^−^^1^
*c* = 11.0438 (4) Å	*T* = 296 K
β = 101.899 (3)°	Prism, colourless
*V* = 2096.96 (14) Å^3^	0.53 × 0.39 × 0.18 mm
*Z* = 4	

### Data collection

**Table:**

Stoe IPDS 2 diffractometer	4352 independent reflections
Radiation source: sealed X-ray tube, 12 x 0.4 mm long-fine focus	3699 reflections with *I* > 2σ(*I*)
plane graphite	*R*_int_ = 0.042
Detector resolution: 6.67 pixels mm^-1^	θ_max_ = 26.5°, θ_min_ = 1.7°
ω scans	*h* = −15→15
Absorption correction: integration (*X-RED32*; Stoe & Cie, 2002)	*k* = −20→20
*T*_min_ = 0.880, *T*_max_ = 0.952	*l* = −13→13
24440 measured reflections	

### Refinement

**Table:**

Refinement on *F*^2^	Primary atom site location: structure-invariant direct methods
Least-squares matrix: full	Secondary atom site location: difference Fourier map
*R*[*F*^2^ > 2σ(*F*^2^)] = 0.040	Hydrogen site location: inferred from neighbouring sites
*wR*(*F*^2^) = 0.121	H-atom parameters constrained
*S* = 1.05	*w* = 1/[σ^2^(*F*_o_^2^) + (0.0658*P*)^2^ + 0.4583*P*] where *P* = (*F*_o_^2^ + 2*F*_c_^2^)/3
4352 reflections	(Δ/σ)_max_ = 0.001
266 parameters	Δρ_max_ = 0.45 e Å^−^^3^
6 restraints	Δρ_min_ = −0.24 e Å^−^^3^

### Special details

**Table:**

Geometry. Bond distances, angles *etc*. have been calculated using the rounded fractional coordinates. All su's are estimated from the variances of the (full) variance-covariance matrix. The cell e.s.d.'s are taken into account in the estimation of distances, angles and torsion angles
Refinement. Refinement on *F*^2^ for ALL reflections except those flagged by the user for potential systematic errors. Weighted *R*-factors *wR* and all goodnesses of fit *S* are based on *F*^2^, conventional *R*-factors *R* are based on *F*, with *F* set to zero for negative *F*^2^. The observed criterion of *F*^2^ > σ(*F*^2^) is used only for calculating -*R*-factor-obs *etc*. and is not relevant to the choice of reflections for refinement. *R*-factors based on *F*^2^ are statistically about twice as large as those based on *F*, and *R*-factors based on ALL data will be even larger.

### Fractional atomic coordinates and isotropic or equivalent isotropic displacement parameters (Å^2^)

**Table:**

	*x*	*y*	*z*	*U*_iso_*/*U*_eq_	Occ. (<1)
S1	0.85182 (4)	0.53595 (3)	0.83953 (4)	0.0541 (2)	
S2	0.61011 (4)	0.47743 (3)	0.81372 (5)	0.0579 (2)	
O1	0.38272 (11)	0.58370 (7)	0.51187 (12)	0.0515 (4)	
O2	0.75522 (13)	0.67788 (8)	0.66272 (13)	0.0642 (5)	
N1	0.38933 (13)	0.70694 (8)	0.40565 (14)	0.0506 (5)	
N2	0.47420 (14)	0.76279 (9)	0.38716 (15)	0.0545 (5)	
N3	0.63560 (12)	0.58980 (8)	0.53913 (13)	0.0462 (4)	
N4	0.74425 (13)	0.52899 (10)	1.02011 (14)	0.0531 (5)	
C1	0.27430 (17)	0.73286 (11)	0.38683 (18)	0.0525 (6)	
C2	0.2427 (2)	0.79527 (14)	0.4585 (2)	0.0742 (8)	
C3	0.1300 (3)	0.81841 (17)	0.4376 (3)	0.0888 (10)	
C4	0.0505 (2)	0.78017 (16)	0.3476 (3)	0.0879 (10)	
C5	0.0838 (2)	0.71980 (17)	0.2760 (3)	0.0915 (10)	
C6	0.1959 (2)	0.69566 (14)	0.2953 (2)	0.0725 (8)	
C7	0.43691 (15)	0.63835 (9)	0.47052 (15)	0.0435 (5)	
C8	0.55651 (15)	0.64845 (10)	0.48024 (16)	0.0443 (5)	
C9	0.57593 (17)	0.72163 (11)	0.42721 (17)	0.0507 (6)	
C10	0.6835 (2)	0.75840 (14)	0.4081 (2)	0.0725 (8)	
C11	0.4516 (2)	0.80451 (14)	0.2664 (2)	0.0764 (9)	
C12	0.72748 (15)	0.60750 (11)	0.62738 (16)	0.0463 (5)	
C13	0.79952 (16)	0.53189 (12)	0.67347 (17)	0.0505 (6)	
C14	0.9026 (2)	0.52810 (19)	0.6130 (2)	0.0815 (9)	
C15	0.72987 (14)	0.51382 (10)	0.89982 (16)	0.0457 (5)	
C16	0.6528 (2)	0.51755 (19)	1.0884 (2)	0.0771 (9)	
C17B	0.7181 (10)	0.5279 (8)	1.2222 (8)	0.098 (3)	0.630 (18)
C18B	0.8057 (7)	0.5936 (4)	1.2131 (7)	0.0721 (19)	0.630 (18)
C19	0.84668 (18)	0.56364 (16)	1.10045 (19)	0.0664 (7)	
C18A	0.8177 (11)	0.5526 (18)	1.2286 (11)	0.104 (6)	0.370 (18)
C17A	0.6894 (13)	0.5621 (18)	1.2098 (17)	0.122 (7)	0.370 (18)
H3	0.10750	0.86050	0.48510	0.1070*	
H2	0.29600	0.82120	0.51960	0.0890*	
H5	0.03060	0.69470	0.21370	0.1100*	
H6	0.21810	0.65430	0.24640	0.0870*	
H10A	0.74570	0.72390	0.44680	0.1090*	
H10B	0.69250	0.81290	0.44410	0.1090*	
H10C	0.68210	0.76220	0.32110	0.1090*	
H3A	0.62460	0.53870	0.51720	0.0550*	
H4	−0.02530	0.79540	0.33560	0.1050*	
H11C	0.45700	0.76490	0.20290	0.1150*	
H13	0.75410	0.48140	0.65250	0.0600*	
H14A	0.87790	0.52970	0.52470	0.1220*	
H14B	0.94340	0.47740	0.63660	0.1220*	
H14C	0.95110	0.57480	0.63980	0.1220*	
H16A	0.64130	0.45890	1.10220	0.0930*	
H16B	0.58250	0.54080	1.04220	0.0930*	
H17C	0.66830	0.54620	1.27560	0.1170*	0.630 (18)
H17D	0.75400	0.47620	1.25410	0.1170*	0.630 (18)
H18C	0.77220	0.64850	1.20040	0.0860*	0.630 (18)
H18D	0.86640	0.59410	1.28610	0.0860*	0.630 (18)
H19A	0.85720	0.62170	1.08210	0.0800*	
H19B	0.91390	0.53260	1.09330	0.0800*	
H11A	0.50620	0.84800	0.26650	0.1150*	
H11B	0.37690	0.82800	0.25090	0.1150*	
H17A	0.66000	0.53540	1.27550	0.1450*	0.370 (18)
H17B	0.66660	0.62000	1.20390	0.1450*	0.370 (18)
H18A	0.84120	0.49810	1.26190	0.1240*	0.370 (18)
H18B	0.85580	0.59450	1.28560	0.1240*	0.370 (18)

### Atomic displacement parameters (Å^2^)

**Table:**

	*U*^11^	*U*^22^	*U*^33^	*U*^12^	*U*^13^	*U*^23^
S1	0.0386 (2)	0.0738 (3)	0.0497 (3)	−0.0019 (2)	0.0086 (2)	0.0032 (2)
S2	0.0453 (3)	0.0736 (3)	0.0553 (3)	−0.0103 (2)	0.0113 (2)	−0.0081 (2)
O1	0.0532 (7)	0.0410 (6)	0.0591 (7)	−0.0065 (5)	0.0088 (6)	0.0045 (5)
O2	0.0728 (9)	0.0548 (8)	0.0604 (8)	−0.0047 (7)	0.0034 (7)	−0.0123 (6)
N1	0.0549 (9)	0.0382 (7)	0.0559 (9)	0.0007 (6)	0.0053 (7)	0.0051 (6)
N2	0.0667 (10)	0.0407 (7)	0.0561 (9)	−0.0019 (7)	0.0129 (7)	0.0111 (6)
N3	0.0497 (8)	0.0385 (7)	0.0494 (8)	−0.0034 (6)	0.0078 (6)	0.0021 (6)
N4	0.0438 (8)	0.0687 (10)	0.0467 (8)	−0.0023 (7)	0.0091 (6)	−0.0025 (7)
C1	0.0595 (11)	0.0402 (8)	0.0557 (10)	0.0066 (8)	0.0067 (8)	0.0033 (7)
C2	0.0799 (16)	0.0648 (13)	0.0736 (14)	0.0117 (11)	0.0059 (12)	−0.0144 (11)
C3	0.0901 (19)	0.0742 (15)	0.103 (2)	0.0279 (14)	0.0222 (16)	−0.0119 (14)
C4	0.0652 (15)	0.0737 (15)	0.123 (2)	0.0174 (12)	0.0150 (15)	0.0054 (15)
C5	0.0669 (15)	0.0819 (16)	0.115 (2)	0.0017 (13)	−0.0059 (15)	−0.0198 (16)
C6	0.0668 (14)	0.0618 (12)	0.0839 (16)	0.0044 (10)	0.0037 (11)	−0.0208 (11)
C7	0.0534 (10)	0.0348 (7)	0.0407 (8)	−0.0024 (7)	0.0059 (7)	−0.0027 (6)
C8	0.0535 (10)	0.0382 (8)	0.0411 (8)	−0.0032 (7)	0.0099 (7)	0.0004 (6)
C9	0.0630 (11)	0.0436 (8)	0.0476 (10)	−0.0050 (8)	0.0160 (8)	0.0020 (7)
C10	0.0752 (15)	0.0657 (12)	0.0816 (15)	−0.0141 (11)	0.0279 (12)	0.0143 (11)
C11	0.0974 (18)	0.0623 (12)	0.0672 (14)	−0.0013 (12)	0.0120 (12)	0.0260 (11)
C12	0.0479 (9)	0.0501 (9)	0.0430 (9)	−0.0038 (7)	0.0145 (7)	−0.0036 (7)
C13	0.0458 (9)	0.0579 (10)	0.0485 (10)	0.0033 (8)	0.0117 (7)	−0.0015 (8)
C14	0.0631 (14)	0.121 (2)	0.0668 (14)	0.0209 (13)	0.0285 (11)	0.0014 (13)
C15	0.0413 (9)	0.0462 (8)	0.0499 (10)	0.0036 (7)	0.0099 (7)	0.0010 (7)
C16	0.0631 (14)	0.118 (2)	0.0555 (12)	−0.0157 (13)	0.0243 (10)	−0.0082 (12)
C17B	0.090 (7)	0.152 (7)	0.057 (3)	−0.016 (5)	0.030 (3)	−0.010 (3)
C18B	0.071 (3)	0.093 (4)	0.049 (3)	0.002 (3)	0.005 (2)	−0.014 (2)
C19	0.0493 (11)	0.0894 (15)	0.0565 (12)	−0.0004 (10)	0.0017 (9)	−0.0107 (11)
C18A	0.069 (6)	0.182 (17)	0.056 (5)	0.008 (9)	0.005 (4)	−0.005 (8)
C17A	0.058 (6)	0.23 (2)	0.082 (8)	−0.021 (9)	0.025 (5)	−0.069 (12)

### Geometric parameters (Å, °)

**Table:**

S1—C13	1.8134 (19)	C17B—C18B	1.512 (15)
S1—C15	1.7717 (18)	C18A—C19	1.537 (13)
S2—C15	1.6637 (18)	C18B—C19	1.509 (8)
O1—C7	1.238 (2)	C2—H2	0.9300
O2—C12	1.221 (2)	C3—H3	0.9300
N1—N2	1.408 (2)	C4—H4	0.9300
N1—C1	1.423 (3)	C5—H5	0.9300
N1—C7	1.375 (2)	C6—H6	0.9300
N2—C9	1.384 (3)	C10—H10A	0.9600
N2—C11	1.467 (3)	C10—H10B	0.9600
N3—C8	1.403 (2)	C10—H10C	0.9600
N3—C12	1.347 (2)	C11—H11A	0.9600
N4—C15	1.326 (2)	C11—H11B	0.9600
N4—C16	1.471 (3)	C11—H11C	0.9600
N4—C19	1.474 (3)	C13—H13	0.9800
N3—H3A	0.8600	C14—H14A	0.9600
C1—C2	1.380 (3)	C14—H14B	0.9600
C1—C6	1.371 (3)	C14—H14C	0.9600
C2—C3	1.383 (4)	C16—H16A	0.9700
C3—C4	1.376 (4)	C16—H16B	0.9700
C4—C5	1.364 (4)	C17A—H17B	0.9700
C5—C6	1.381 (4)	C17A—H17A	0.9700
C7—C8	1.434 (3)	C17B—H17D	0.9700
C8—C9	1.356 (2)	C17B—H17C	0.9700
C9—C10	1.480 (3)	C18A—H18B	0.9700
C12—C13	1.520 (3)	C18A—H18A	0.9700
C13—C14	1.529 (3)	C18B—H18D	0.9700
C16—C17A	1.50 (2)	C18B—H18C	0.9700
C16—C17B	1.534 (9)	C19—H19A	0.9700
C17A—C18A	1.53 (2)	C19—H19B	0.9700
			
S1···O2	3.0730 (14)	C10···H11A	2.7800
S2···C16^i^	3.557 (2)	C11···H10C	2.8100
S2···C12	3.4378 (19)	C12···H10A	2.7800
S2···C7^ii^	3.5929 (17)	C15···H10B^vi^	2.8800
S2···C17A^i^	3.625 (17)	C16···H16B^i^	3.0500
S1···H19B	2.7500	C18A···H14B^iv^	3.0000
S1···H19A	3.0000	C19···H10B^vi^	3.0100
S1···H4^iii^	3.1000	H2···S2^viii^	3.1800
S1···H19B^iv^	2.9800	H3A···H13	2.1400
S2···H13	2.7300	H3A···O1^ii^	1.9900
S2···H2^v^	3.1800	H3A···C7^ii^	2.9500
S2···H16B	2.8000	H4···S1^ix^	3.1000
S2···H6^ii^	3.1300	H5···H19A^x^	2.5700
S2···H16B^i^	3.0900	H6···H13^ii^	2.4400
S2···H16A	3.1400	H6···S2^ii^	3.1300
S2···H11A^vi^	3.0800	H10A···O2	2.4800
O1···N3	3.005 (2)	H10A···N3	2.8300
O1···N3^ii^	2.8463 (17)	H10A···C12	2.7800
O1···C13^ii^	3.260 (2)	H10B···N4^vii^	2.7100
O1···C11^vi^	3.296 (3)	H10B···H19A^vii^	2.4800
O2···C10^vi^	3.180 (3)	H10B···C19^vii^	3.0100
O2···C9	3.102 (2)	H10B···C15^vii^	2.8800
O2···S1	3.0730 (14)	H10C···H11A	2.5000
O2···C10	3.054 (3)	H10C···O2^vii^	2.3200
O1···H13^ii^	2.4300	H10C···C11	2.8100
O1···H3A^ii^	1.9900	H11A···S2^vii^	3.0800
O2···H10A	2.4800	H11A···C10	2.7800
O2···H10C^vi^	2.3200	H11A···H10C	2.5000
O2···H18C^vii^	2.8200	H11B···C1	2.6300
N3···O1	3.005 (2)	H11C···C7^vii^	2.9700
N3···O1^ii^	2.8463 (17)	H13···H3A	2.1400
N3···H10A	2.8300	H13···S2	2.7300
N4···H10B^vi^	2.7100	H13···C7^ii^	3.0900
C7···S2^ii^	3.5929 (17)	H13···O1^ii^	2.4300
C7···C11^vi^	3.364 (3)	H13···C6^ii^	2.9400
C9···O2	3.102 (2)	H13···H6^ii^	2.4400
C10···O2	3.054 (3)	H14B···H18D^iv^	2.5500
C10···C12	3.392 (3)	H14B···C18A^iv^	3.0000
C10···O2^vii^	3.180 (3)	H16A···S2	3.1400
C11···O1^vii^	3.296 (3)	H16B···S2	2.8000
C11···C7^vii^	3.364 (3)	H16B···H16B^i^	2.4100
C12···C10	3.392 (3)	H16B···S2^i^	3.0900
C12···S2	3.4378 (19)	H16B···C16^i^	3.0500
C13···O1^ii^	3.260 (2)	H18C···O2^vi^	2.8200
C16···S2^i^	3.557 (2)	H18D···H14B^iv^	2.5500
C17A···S2^i^	3.625 (17)	H19A···H5^xi^	2.5700
C1···H11B	2.6300	H19A···S1	3.0000
C6···H13^ii^	2.9400	H19A···H10B^vi^	2.4800
C7···H13^ii^	3.0900	H19B···S1^iv^	2.9800
C7···H11C^vi^	2.9700	H19B···S1	2.7500
C7···H3A^ii^	2.9500		
			
C13—S1—C15	103.30 (9)	C5—C6—H6	120.00
N2—N1—C1	120.56 (13)	C9—C10—H10A	109.00
N2—N1—C7	110.45 (15)	C9—C10—H10B	109.00
C1—N1—C7	126.95 (15)	C9—C10—H10C	109.00
N1—N2—C9	105.78 (14)	H10A—C10—H10B	109.00
N1—N2—C11	114.77 (16)	H10A—C10—H10C	109.00
C9—N2—C11	119.70 (17)	H10B—C10—H10C	110.00
C8—N3—C12	124.97 (14)	N2—C11—H11A	109.00
C15—N4—C16	122.24 (16)	N2—C11—H11B	110.00
C15—N4—C19	126.35 (16)	N2—C11—H11C	109.00
C16—N4—C19	111.29 (16)	H11A—C11—H11B	109.00
C8—N3—H3A	117.00	H11A—C11—H11C	110.00
C12—N3—H3A	118.00	H11B—C11—H11C	110.00
C2—C1—C6	120.7 (2)	S1—C13—H13	109.00
N1—C1—C2	120.40 (18)	C12—C13—H13	109.00
N1—C1—C6	118.93 (18)	C14—C13—H13	109.00
C1—C2—C3	118.6 (2)	C13—C14—H14A	109.00
C2—C3—C4	121.0 (3)	C13—C14—H14B	109.00
C3—C4—C5	119.5 (3)	C13—C14—H14C	109.00
C4—C5—C6	120.5 (3)	H14A—C14—H14B	109.00
C1—C6—C5	119.7 (2)	H14A—C14—H14C	110.00
O1—C7—N1	124.51 (17)	H14B—C14—H14C	110.00
O1—C7—C8	130.76 (15)	N4—C16—H16A	110.00
N1—C7—C8	104.70 (14)	N4—C16—H16B	110.00
N3—C8—C7	122.44 (14)	C17B—C16—H16A	91.00
C7—C8—C9	109.07 (16)	C17B—C16—H16B	133.00
N3—C8—C9	128.48 (17)	H16A—C16—H16B	109.00
N2—C9—C10	120.60 (17)	C17A—C16—H16A	110.00
N2—C9—C8	109.40 (17)	C17A—C16—H16B	110.00
C8—C9—C10	130.00 (19)	C16—C17A—H17B	112.00
O2—C12—C13	122.30 (17)	C18A—C17A—H17A	112.00
O2—C12—N3	123.88 (17)	C16—C17A—H17A	112.00
N3—C12—C13	113.68 (15)	H17A—C17A—H17B	109.00
C12—C13—C14	110.24 (17)	C18A—C17A—H17B	112.00
S1—C13—C14	107.36 (14)	C16—C17B—H17D	111.00
S1—C13—C12	111.44 (13)	C16—C17B—H17C	111.00
S1—C15—S2	123.13 (10)	C18B—C17B—H17C	111.00
S1—C15—N4	113.09 (13)	C18B—C17B—H17D	111.00
S2—C15—N4	123.78 (14)	H17C—C17B—H17D	109.00
N4—C16—C17B	100.9 (5)	C19—C18A—H18A	110.00
N4—C16—C17A	106.5 (7)	C19—C18A—H18B	110.00
C16—C17A—C18A	100.2 (14)	C17A—C18A—H18A	111.00
C16—C17B—C18B	103.8 (7)	C17A—C18A—H18B	110.00
C17A—C18A—C19	106.3 (10)	H18A—C18A—H18B	109.00
C17B—C18B—C19	100.6 (6)	C17B—C18B—H18C	112.00
N4—C19—C18B	103.9 (3)	C19—C18B—H18D	112.00
N4—C19—C18A	100.9 (6)	C17B—C18B—H18D	112.00
C1—C2—H2	121.00	C19—C18B—H18C	112.00
C3—C2—H2	121.00	H18C—C18B—H18D	109.00
C2—C3—H3	120.00	C18B—C19—H19A	87.00
C4—C3—H3	119.00	C18B—C19—H19B	131.00
C3—C4—H4	120.00	C18A—C19—H19A	112.00
C5—C4—H4	120.00	C18A—C19—H19B	112.00
C4—C5—H5	120.00	H19A—C19—H19B	109.00
C6—C5—H5	120.00	N4—C19—H19A	112.00
C1—C6—H6	120.00	N4—C19—H19B	112.00
			
C15—S1—C13—C14	−165.83 (16)	C15—N4—C19—C18B	162.2 (3)
C13—S1—C15—S2	12.21 (13)	C16—N4—C15—S2	−2.2 (3)
C15—S1—C13—C12	73.36 (14)	C19—N4—C15—S2	−177.92 (16)
C13—S1—C15—N4	−168.00 (13)	C15—N4—C16—C17B	170.4 (5)
C1—N1—N2—C9	172.98 (16)	C2—C1—C6—C5	−1.2 (3)
C1—N1—N2—C11	−52.8 (2)	N1—C1—C2—C3	−179.8 (2)
N2—N1—C1—C6	116.6 (2)	N1—C1—C6—C5	179.8 (2)
C7—N1—C1—C6	−81.3 (2)	C6—C1—C2—C3	1.2 (3)
N2—N1—C1—C2	−62.5 (2)	C1—C2—C3—C4	0.2 (4)
C7—N1—N2—C9	8.12 (19)	C2—C3—C4—C5	−1.5 (4)
C7—N1—N2—C11	142.34 (16)	C3—C4—C5—C6	1.5 (4)
C1—N1—C7—O1	7.9 (3)	C4—C5—C6—C1	−0.2 (4)
C7—N1—C1—C2	99.7 (2)	O1—C7—C8—N3	3.3 (3)
N2—N1—C7—O1	171.58 (15)	N1—C7—C8—C9	2.35 (19)
N2—N1—C7—C8	−6.45 (18)	N1—C7—C8—N3	−178.88 (15)
C1—N1—C7—C8	−170.10 (16)	O1—C7—C8—C9	−175.50 (18)
N1—N2—C9—C8	−6.5 (2)	C7—C8—C9—C10	−176.69 (19)
C11—N2—C9—C10	41.5 (3)	C7—C8—C9—N2	2.7 (2)
C11—N2—C9—C8	−137.95 (18)	N3—C8—C9—C10	4.6 (3)
N1—N2—C9—C10	172.95 (16)	N3—C8—C9—N2	−176.03 (16)
C8—N3—C12—O2	−3.3 (3)	O2—C12—C13—C14	−75.2 (2)
C8—N3—C12—C13	−179.08 (16)	O2—C12—C13—S1	43.9 (2)
C12—N3—C8—C9	49.4 (3)	N3—C12—C13—S1	−140.25 (14)
C12—N3—C8—C7	−129.09 (18)	N3—C12—C13—C14	100.64 (19)
C16—N4—C19—C18B	−13.9 (3)	N4—C16—C17B—C18B	35.6 (8)
C19—N4—C16—C17B	−13.3 (5)	C16—C17B—C18B—C19	−44.4 (8)
C19—N4—C15—S1	2.3 (2)	C17B—C18B—C19—N4	35.3 (6)
C16—N4—C15—S1	178.00 (17)		

Symmetry codes: (i) −*x*+1, −*y*+1, −*z*+2; (ii) −*x*+1, −*y*+1, −*z*+1; (iii) *x*+1, −*y*+3/2, *z*+1/2; (iv) −*x*+2, −*y*+1, −*z*+2; (v) −*x*+1, *y*−1/2, −*z*+3/2; (vi) *x*, −*y*+3/2, *z*+1/2; (vii) *x*, −*y*+3/2, *z*−1/2; (viii) −*x*+1, *y*+1/2, −*z*+3/2; (ix) *x*−1, −*y*+3/2, *z*−1/2; (x) *x*−1, *y*, *z*−1; (xi) *x*+1, *y*, *z*+1.

### Hydrogen-bond geometry (Å, °)

**Table:**

*D*—H···*A*	*D*—H	H···*A*	*D*···*A*	*D*—H···*A*
N3—H3A···O1^ii^	0.86	1.99	2.8463 (17)	171
C10—H10A···O2	0.96	2.48	3.054 (3)	119
C10—H10C···O2^vii^	0.96	2.32	3.180 (3)	148
C13—H13···S2	0.98	2.73	3.138 (2)	105
C13—H13···O1^ii^	0.98	2.43	3.260 (2)	143

Symmetry codes: (ii) −*x*+1, −*y*+1, −*z*+1; (vii) *x*, −*y*+3/2, *z*−1/2.
